# Histological, histochemical and ultrastructural analysis reveals functional division of the oesophagogastric segment in freshwater tubenose goby *Proterorhinus semilunaris* Heckel, 1837

**DOI:** 10.1007/s00435-014-0250-7

**Published:** 2014-12-11

**Authors:** Katarzyna Wołczuk, Julita Nowakowska, Dariusz Płąchocki, Tomasz Kakareko

**Affiliations:** 1 Department of Zoology of Vertebrates, Nicolaus Copernicus University, Lwowska 1, 87-100 Toruń, Poland; 2 Laboratory of Electron and Confocal Microscopy, University of Warsaw, Miecznikowa 1, Warsaw, Poland; 3 Department of Hydrobiology, Nicolaus Copernicus University, Lwowska 1, 87-100 Toruń, Poland

**Keywords:** *Proterorhinus semilunaris*, Oesophagus, Stomach, Gastric gland, Oxynticopeptic cell, Proliferating cells

## Abstract

Histological and histochemical features of the oesophagogastric segment of the alimentary canal as well as ultrastructure of gastric gland cells of freshwater tubenose goby *Proterorhinus semilunaris* were examined. The studies revealed that despite the lack of anatomical distinction, the oesophagogastric segment is histologically divided into the oesophagus, oesogaster and stomach, which provides evidence for the functional compartmentation of this organ. The oesophagus was characterised by the presence of numerous goblet cells secreting mainly a mixture of neutral and acid mucopolysaccharides. In the stomach, the apical zone of the surface epithelial cells contained neutral mucopolysaccharides. Numerous proliferating cells were scattered throughout the surface epithelium. In the lamina propria of the stomach, a well-developed layer of gastric glands was observed. The glands were of the alveolar type and occupied nearly the entire length of the stomach except the pyloric region. The gastric gland cells were varied into light and dark; however, their ultrastructure was identical. All cells had numerous mitochondria and a well-developed tubulovesicular system typical for the oxynticopeptic cells, but pepsinogen granules were not present in the cytoplasm of these cells. These findings contribute new evidence to literature reports that not all gobiid fish are stomachless. Moreover, they suggest higher adaptation of the species to utilise protein-rich food compared to stomachless fish, and its ability to adjust the alimentary canal quickly to changing diet. How this may facilitate establishment of *P. semilunaris* in invaded environments remains an open question.

## Introduction

Gobiidae is one of the largest families of the acanthomorph fish including over 1,950 species inhabiting marine, brackish, as well as fresh waters of the moderate and tropical zones (Nelson 2006; Thacker 2011; Thacker and Roje 2011). It includes herbivores, omnivores as well as carnivores (Geevarghese 1983; Wu et al. 2010). Varied food preferences as well as environmental conditions in which the Gobiidae live contributed to the development of a number of adaptation features in the structure of their digestive system (Geevarghese 1983; Kobegenova and Dzhumaliev 1991). These fish have one feature in common though: they lack a well-developed stomach (Geevarghese 1983; Kobegenova and Dzhumaliev 1991). This anatomical feature of the fish alimentary canal led to confusion among researchers as many regarded this fish family as stomachless (Barton 2007; Jaroszewska et al. 2008). Although not all researchers share this opinion (Geevarghese 1983; Hur et al. 2005; Wu et al. 2010), there is still lack of clear evidence concerning histological and physiological aspects of the oesophagogastric segment that not all Gobiidae are stomachless. The majority of conclusions here were drawn on the basis of the results of anatomical research (Geevarghese 1983; Pogoreutz and Ahnelt 2014; Wilson and Castro 2011). The data from the literature concerning the histology of the alimentary canal of the Gobiidae are limited. Until the present moment, the histology of several Gobiidae species only has been studied, such as *Babka gymnotrachelus*, *Rhinogobius giurinus,* *Neogobius ratan*, *Neogobius melanostomus*, *Mesogobius batrachocephalus* and *Neogobius fluviatilis* (Kobegenova and Dzhumaliev 1991; Hur et al. 2005; Jaroszewska et al. 2008), and the study was not always based on appropriate sample size (Kobegenova and Dzhumaliev 1991). In the light of the foregoing, it seemed interesting to commence research into whether or not the stomach is present in Gobiidae. Particularly, it seemed pertinent to study further this aspect in Ponto-Caspian gobiids. These fish have invaded or expanded their range in European waters (Grabowska et al. 2008; Roche et al. 2013), and it is worthwhile to know if they possess any peculiarities in the structure and/or function of the alimentary tract that might facilitate their establishment in novel ecosystems.

One of the gobiid species, freshwater tubenose goby *Proterorhinus semilunaris* (Heckel, 1837), considerably extended its geographical range in the early twentieth century, and it is regarded as an invasive species in the inland waters of Central Europe including Poland (Grabowska et al. 2008; Adámek et al. 2010). According to the researchers, the features behind the success in colonising new areas include high adaptability and feeding opportunism of *P. semilunaris* (Adámek et al. 2010; Všeticková et al. 2014). This proven feeding plasticity may be reflected in the structure and function of the alimentary tract of *P. semilunaris;* however, such studies have not been conducted.

In this study, we determined the morphological features of the oesophagogastric segment of *P. semilunaris* based on the histological, histochemical and ultrastructural analysis, in order to provide evidence for a functional division (or lack thereof) of this structure. It was expected that this segment does not have both an anatomical distinction and histological regionalisation, confirming that *P. semilunaris* is a stomachless fish species. On the other hand, it was hypothesised that the substantial feeding plasticity of this species may be associated with morphological and functional features of the oesophagogastric segment, in particular with properties of epithelial cells which might be significantly favourable for becoming established of *P. semilunaris* in newly invaded environments.

## Materials and methods

### Animals

Fish were obtained with support of Dr Katarzyna Mierzejewska from Warmia and Mazury University in Olsztyn, Poland. Ethical approvals were received from the Local Committee, Warmia and Mazury University in Olsztyn, Poland, Resolution 108/2010. Adult specimens of freshwater tubenose goby *P. semilunaris* were collected in June 2011 in Włocławek Reservoir associated with the lower Vistula River in central Poland. In the study, twelve specimens were used. The fish were euthanised with benzocaine (50 mg/l), and the alimentary canal was removed from the fish abdomen.

### Light microscopy

The alimentary canals of ten fish were fixed for 24 h in 10 % neutral buffered formalin, then rinsed with running water and divided into two sections of which the oesophagogastric segment (Geevarghese 1983) was used for examination. This part of the alimentary canal was dehydrated in ethanol, embedded in paraffin and cut transversely (six specimens) and longitudinally (four specimens) using the Microm HM 355 microtome. Serial samples 5 µm thick were stained routinely with haematoxylin–eosin (H–E) and histochemically with periodic acid Schiff (PAS) and alcian blue (AB) at pH 2.5 (AB-PAS) for neutral and acid mucopolysaccharides. To label proliferating epithelial cells in the oesophagus and stomach, the PCNA immunostaining was performed using the labelled streptavidin biotin (LSAB) method protocol, described by DAKO (LSAB + HRP Kit, DAKO). The slides were dewaxed, using xylene and transferred to alcohol. Then, they were placed in target retrieval solutions (pH = 6.0; TRS, Dako) and heated in a microwave oven (790 W) for 15 min to expose antigens. Endogenous peroxidase activity was blocked by incubating the section with 0.3 % H_2_O_2_ in methanol for 15 min. After washing with PBS, the slides were incubated at 20 °C for 1 h with mouse anti-human monoclonal antibody PCNA (PC 10, DAKO, dilution 1:90). The reaction products were visualised with diaminobenzidine DAB (DAKO).

The prepared slides were used for the histological analysis and measurement of the individual thicknesses of the layers making up the wall of the oesophagus, oesogaster (transitional zone or oesophagogastric junction) and stomach. Using the Olympus CX21 microscope fitted with a calibrated eyepiece (with the accuracy of 1.7 µm and linear magnification of 128×), the thickness of the epithelium, lamina propria of mucosa, muscularis and serosa were measured, and additionally the height and width of the alveolar gland, occurring in the stomach region. For each of these parameters, 20 measurements were taken of which an average was calculated. The results were presented as mean ± SD values.

Photographs were taken using an Olympus 500 camera and an Olympus CX21 light microscope.

### Transmission electron microscopy

Small fragments of two stomachs were fixed for 4 h with 2.5 % glutaraldehyde in 0.1 M phosphate buffer containing 5 % sucrase. Next, the samples were postfixed in 1 % OsO_4_ in ddH_2_O for 1 h on ice, washed three times in ddH_2_O, dehydrated and embedded in pure epon resin. The 60-nm sections were prepared and contrasted with uranyl acetate and lead citrate according to Reynollds (1983) and were examined on LEO 912AB transmission electron microscope produced by Zeiss. Images were captured with the Slow Scane CCD (Proscane) using EsiVision Pro 3.2 software (Soft Imaging Systems GmbH) and were used to describe the ultrastructure of the cells making up the gastric glands.

## Results

The body mass of specimens of *P. semilunaris* was 1.8 ± 0.6 g, and the standard body length (*L*_s_) was 53.8 ± 2.4 mm.

**Fig. 1:**
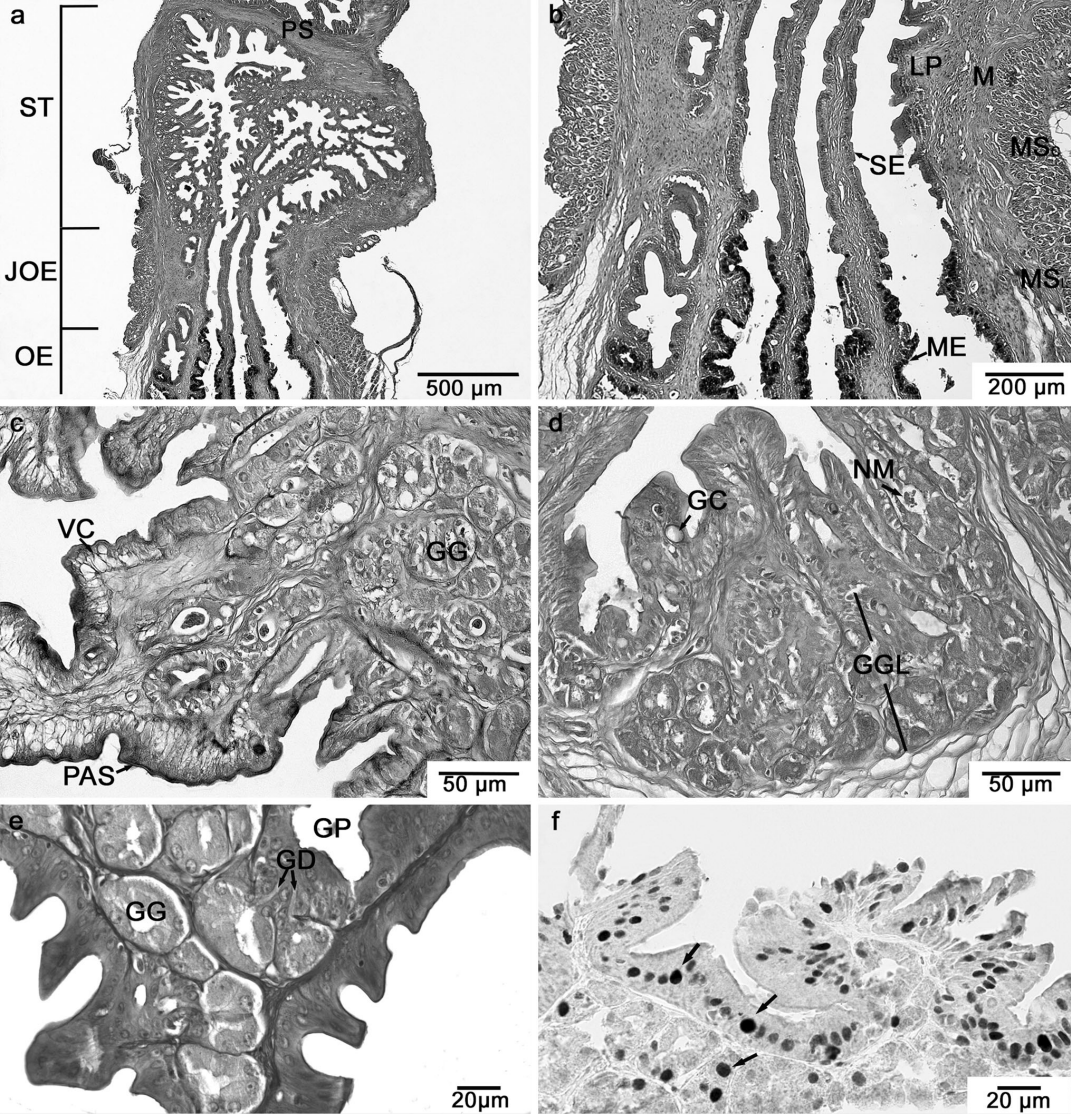
Histology of oesophagogastric segment in the freshwater tubenose goby *P. semilunaris* reveals functional division. **a** Longitudinal section through the oesophagus (*OE*), oesogaster (*JOE*), stomach (*ST*) and pyloric sphincter (*PS*); AB-PAS staining. **b** Longitudinal section through the oesogaster showing abrupt transition from stratified squamous epithelium of the oesophagus (*ME*) to a simple columnar epithelium of the stomach (*SE*) (*LP* lamina propria, *M* smooth muscles, *MS* striated muscles); AB-PAS staining. **c** Transverse section of gastric mucosa showing vacuolated epithelial cells with a PAS-positive apical cytoplasm (PAS) (*GG*, alveolar gastric gland); AB-PAS staining. **d** Transverse section of gastric mucosa showing goblet cell (*GC*) in the epithelium, neutral mucopolysaccharides (*NM*) in the lumen of alveolar gastric gland and well-developed layer of alveolar gastric glands (*GG*); AB-PAS staining. **e** Transverse section of gastric mucosa showing alveolar gastric glands (*GG*) with ducts (*GD*) opening into the gastric pits (*GP*); H–E staining. **f** Transverse section of gastric mucosa showing proliferating cells in the epithelium and in the neck region of gastric glands (*arrows*); PCNA immunostaining

The oesophagogastric segment of the freshwater tubenose goby was not varied anatomically into oesophagus and stomach. A clear separation of the stomach from the neighbouring section (the oesophagus and the small intestine) was possible only on the basis of histological analysis (Fig. 1a). The thickness (µm) of tissue layers forming the wall of oesophagogastric segment in *P. semilunaris* are presented in Table 1.

**Table 1:** Thickness (µm) of tissue layers forming the wall of oesophagogastric segment in the freshwater tubenose goby *Proterorhinus semilunaris*

Regions	Mucosa			Muscularis		Serosa
	Epithelium	Lamina propria				
		Connective tissue layer	Gastric glands layer	Inner layer	Outer layer	
Oesophagus	24.1 ± 3.2	15.7 ± 2.9		73.8 ± 16.4	58.5 ± 23.5	5.1 ± 1.7
Stomach	20.2 ± 3.5	21.1 ± 8.1	54.4 ± 6.6	24.4 ± 5.8	26.2 ± 7.0	2.5 ± 1.7

### Oesophagus

The wall of the oesophagus was made up of mucosa, muscularis and serosa. The mucosa formed longitudinal branching folds including primary and secondary folds, and the height of these folds was the smallest in the initial region of the oesophagus and increased in its distal region. Epithelial lamina of the mucosa was formed by the stratified squamous epithelium. The basal layer of epithelium consisted of the prismatic cells with a round, centrally situated nucleus. The proliferation of these cells took place along the entire length of the epithelium inside and between the folds. Within the epithelium numerous, large goblet cells were observed. They were sac-like structures and occupied nearly the entire thickness of the epithelium. They mainly included positively stained AB-PAS cells which proved the parallel presence of acid and neutral mucopolysaccharides. Less numerous were the AB-positive or PAS-positive cells. The epithelium also included the rodlet cells scattered along the entire length of the oesophagus. Inclusions observed in cytoplasm of rodlet cells were PAS positive. The lamina propria of mucosa was made up of dense connective tissue immediately beneath the epithelium with a slightly looser structure below. It was mainly made up of collagen fibres.

The muscularis was formed by two layers of striated skeletal muscles. The outer layer was formed by the circular organised muscles, and the inner layer was formed by the longitudinally situated bundles of muscle fibres which were present only on the lateral walls of the oesophagus.

The wall of the oesophagus was covered with a very thin serosa (Table 1).

### Oesogaster (transitional zone or oesophagogastric junction)

In this part of the oesophagogastric segment in the microscopic observation, a subtle narrowing was observed allowing the border between the oesophagus and the stomach to be determined. The structure of this region clearly marked the transition of the stratified squamous epithelium of the oesophagus into the simple columnar epithelium of the stomach (Fig. 1b). The cells of the columnar epithelium were tightly packed and had an elongated nucleus situated at the base of the cell. In the epithelium, there were no goblet cells however in the apical zone of the epithelial cells the cytoplasm was PAS positive.

The lamina propria of mucosa was without gastric glands and had a structure typical for the oesophagus (Fig. 1b). In the mucosa, the inner layer of the striated muscles was replaced with the smooth muscles which occurred in the more proximal area of the transition zone. The striated muscles making up the outer layer of the muscularis ended in the distal part of the transition zone. In the same place on the internal side of the smooth muscles with longitudinal orientation, smooth muscles in a circular arrangement were observed.

### Stomach

The stomach was formed by a short bag-like section situated between the oesophagus and the small intestine. The wall of the stomach, similarly to the oesophagus, was composed by the mucosa, muscularis and serosa. The mucosa of the stomach was formed by the longitudinal folds connected with anastomoses. The surface layer of the mucosa was made up of the simple columnar epithelium. In the apical zone of the surface epithelium, the cytoplasm contained PAS-positive granules, which confirmed the presence of neutral mucopolysaccharides. Between the columnar epithelial cells, there were few PAS-positive goblet cells and numerous rodlet cells whose inclusions were PAS positive. In four specimens, the epithelium also contained strongly vacuolated cells (Fig. 1c). The proliferating epithelial cells were scattered across the surface epithelium (Fig. 1d).

Beneath the epithelium, there was lamina propria of mucosa subdivided into a glandular layer and connective tissue layer. Directly beneath the epithelium along nearly the entire length of the stomach (1,870 ± 264 µm) excepting the pyloric region, there was a compact layer of multicellular glands of alveolar type (Fig. 1d). The sizes of each individual alveolus were 35.4 ± 4.3 µm in width and 35.5 ± 4.5 µm in height. The glands were grouped and associated with the common efferent ducts which opened at the bottom of the gastric pits (Fig. 1e). In the lumen of the gland, there was PAS-positive mucus which proved the presence of the neutral mucopolysaccharides (Fig. 1d). However, the gland cells were not AB-PAS positive except for individual inclusions present in the cytoplasm of some cells making up the glands. Few proliferating cells were present in the evacuating part of the gland (Fig. 1f).

**Fig. 2:**
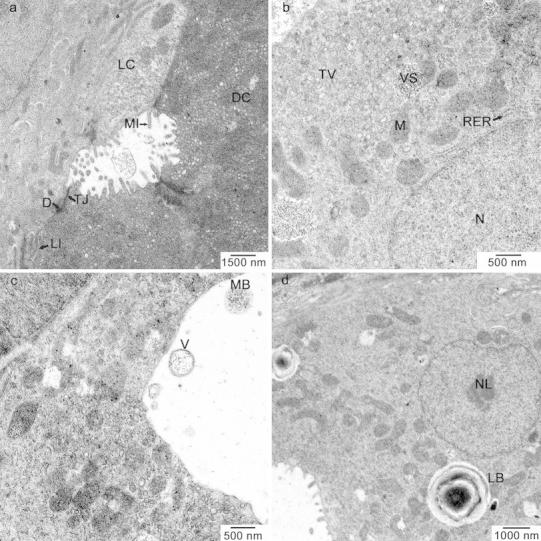
Ultrastructure of gastric gland cells in the freshwater tubenose goby *P. semilunaris*. **a** Electron micrograph of gastric gland showing light (*LC*) and dark (*DC*) cells connected by tight junctions (*TJ*) and desmosomes (*D*), interdigitations (*IL*) in the lateral surface of the cell, microvilli (*MI*) in the luminal surface of the cells. **b** Electron micrograph of gastric gland cell showing well-developed tubulovesicular system (*TV*), numerous mitochondria (*M*), spherical nucleus (*N*) and some vesicular structures containing low-density material (*VS*). **c** Electron micrograph of gastric gland showing multivesicular bodies (*MB*) and vesicles with low-density material (*V*) in the gland lumen. **d** Electron micrograph of gastric gland cell showing nucleus with a distinct nucleolus (*NL*) and vesicles with lamellar bodies (*LB*)

The TEM analysis showed that the glandular cells were varied into light and dark; however, their ultrastructure was identical (Fig. 2a). All cells were trapezoidal in shape with a round nucleus situated at the base of the cell (Fig. 2d). The nucleus was characterised by low density of the nuclear material and a clearly visible nucleolus (Fig. 2d). Next to the nucleus was poorly developed Golgi apparatus and rough endoplasmic reticulum. A considerable area of the supranuclear cytoplasm was occupied by numerous large oval or rod-like mitochondria (Fig. 2b, c, d). Some of the mitochondria were coupled with the vesicular structure containing low-density material (Fig. 2b). In the supranuclear area, a well-developed tubulovesicular network of smooth endoplasmic reticulum was observed (Fig. 2b). Pepsinogen granules were not found, but large vacuoles containing lamellar bodies were occasionally noticed (Fig. 2d). The cell membrane contained numerous microvilli on the luminal surface and formed interdigitations on the lateral part of the cell surface (Fig. 2a) and was smooth in the basal part. The cells were connected with one another through tight junctions and desmosomes (Fig. 2a). In the gland lumen, multivesicular bodies were visible as well as some vesicles including low electron density material (Fig. 2c).

Under the glandular layer, there was a thin layer of connective tissue containing parallel collagen fibres and numerous blood vessels. As in the oesophagus, the muscularis mucosa was not present.

The muscularis of the stomach was composed of two layers of smooth muscles, the inner circular and the outer longitudinal. The total thickness of the muscularis was clearly thinner in comparison with the oesophagus (Table 1). On the border of the stomach and small intestine, there was a pyloric sphincter formed by the thickening of the inner layer of the muscles (Fig. 1a). In this region, the thickness of this layer of muscularis was up to 80 µm.

The serosa of the stomach wall was very thin (Table 1) and made up of mesothelium and loose connective tissue.

## Discussion

The present research showed that even though the oesophagogastric segment of the alimentary canal of *P. semilunaris* does not have an anatomically separate stomach (which was previously shown in other gobiid fishes by Kobegenova and Dzhumaliev 1991 and Jaroszewska et al. 2008), it has a functional stomach which is evident in histological, histochemical and ultrastructural features. This finding changes the view on the stomachless nature of the alimentary canal of Gobiidae, but most of all, it uncovers hidden peculiarities of *P. semilunaris* alimentary canal, showing higher adaptation of the species to utilise protein-rich food compared to stomachless fish, and suggesting its potential to adjust the alimentary canal quickly to changing diet, which may facilitate establishment of the species in dynamic, nutritionally variable environments. But how important the role of the functional stomach is in digestion remains an open question. It is expected to be limited as the capacity of this organ is small, which is associated with the lack of anatomical distinction. Lack of an anatomically separate stomach was previously noted in other members of Gobiidae, which constituted the basis for the classification of this fish family into the group of fish without a stomach (Chao 1973; Grosell 2011). However, the histological structure of the wall of this region in *P. semilunaris* does not seem to confirm this classification. There are two major histological indicators of the existence of the functional stomach that suggest an advantage to this species in the digestion of protein-rich food. These are the presence of the following: (1) goblet cells containing neutral polysaccharides in the apical region of the surface epithelial cells, (2) gastric glands in the mucosa of the stomach area. The indicator of the high adaptation capacity of the oesophagogastric segment of the alimentary canal to variable diet might be the arrangement of proliferating cells of the stomach epithelium as well as the properties of oesophageal mucous cells. The details of these findings are discussed below.

### Surface epithelial cells with neutral polysaccharides

The presence of a neutral polysaccharide in the apical region of the epithelial cells lining the lumen of this region in *P. semilunaris* makes this section similar to the stomach of most fish (Diaz et al. 2008; Leknes 2011; Naguib et al. 2011; Veira-Lopes et al. 2013). These substances may play an important part in the mucosa protection against mechanic injuries during the passage of food and additionally create proper conditions for absorption of disaccharides and short-chain fatty acids (Grau et al. 1992; Murray et al. 1994; Petrinec et al. 2005; Hernández et al. 2009). In *P. semilunaris*, the absorption of easily digestible substances through the cells of surface epithelium seems to be confirmed by the presence of vacuolated cells observed in the surface epithelium of the stomach. It cannot be excluded that the neutral mucus protects the mucosa against the effect of hydrochloric acid and digestive enzymes as is the case in other fish (Grau et al. 1992; Scocco et al. 1996; Morrison and Wright 1999; Naguib et al. 2011); nevertheless, this hypothesis requires verification through further research. In the Gobiidae family, representatives analysed so far no neutral mucous substances were identified on the surface of the epithelium in this region (Kobegenova and Dzhumaliev 1991; Hur et al. 2005; Jaroszewska et al. 2008), which contributed to the assumption that in the stomach of these fish the hydrochloric acid is not produced and that mucosa protection against its effect is not necessary.

### Gastric glands

In fish, the gastric glands take the form of tubes or alveoles separated by the connective tissue bands and the level of development of these structures as well as their location depend on the feeding habits of the species (Murray et al. 1994; Veira-Lopes et al. 2013). In *P. semilunaris*, as in *R. giurinus*, *M. batrachocephalus*, *B. gymnotrachelus*, *N. melanostomus* and *N. ratan* (Kobegenova and Dzhumaliev 1991; Hur et al. 2005; Jaroszewska et al. 2008) in the mucosa of the stomach area alveolar glands were present. They combined to form a dense well-developed layer and occurred along nearly the entire length of this section as described in *M. batrachocephalus* and made it distinct from the glands of other Gobiidae species (Kobegenova and Dzhumaliev 1991; Jaroszewska et al. 2008). This similarity between *P. semilunaris* and *M. batrachcephalus* may be due to the close phylogenetic relationship of these species (Neilson and Stepien 2009).

In the lumen of the gastric glands of *P. semilunaris*, neutral mucous substances were present which were also observed in *B. gymnotrachelus* (Jaroszewska et al. 2008). Jaroszewska et al. (2008) concluded that the mucus is a result of secretive activity of the cells making up the alveolar glands. It was indicated by the presence of PAS-positive substances in some cells of the glands (Jaroszewska et al. 2008). The histochemical research into *P. semilunaris* stomach did not identify the presence of any mucous substances in the gastric gland cells. Furthermore, the ultrastructure analysis of these cells did not confirm any secretive granules included mucus. According to our assumptions, the mucous substances present in the lumen of the glands may be a secretion from the surface epithelial cells of the stomach and/or goblet cells located in the gastric and oesophageal epithelium.

As regards the ultrastructure of the cells making up the glands, it recalls of the structure of the oxynticopeptic cells. The oxynticopeptic cells constitute the main type of secretive cells comprised in the stomach glands of fish as well as amphibians, reptiles and birds (Gargiulo et al. 1997; Gallego-Huidobro and Pastor 1996; Liquori et al. 2000). As numerous research showed, the oxynticopeptic cells bring together the structure and function of chief and parietal cells observed in mammals (Barrington 1957; Naguib et al. 2011). They have also a developed system of the tubulovesicular network of smooth membrane related to secretion of hydrochloric acid and numerous secretive granules and well-developed RER related to the production of pepsinogen (Naguib et al. 2011). In *P. semilunaris*, the glandular cells were characterised by a strong development of the tubulovesicular system and a considerable number of mitochondria present in the entire cytoplasm of the cell; however, the granules of the zymogen were not observed. Glandular cells in tilapia had a similar structure (Wang and Wang 1989; Gargiulo et al. 1997). According to Gargiulo et al. (1997), the ultrastructural organisation of the cells recalls the one present in parietal cells of the mammals which is responsible for secretion of hydrochloric acid, and therefore, he referred to them as oxyntic cells. Also in *P. semilunaris*, the structure of the glandular cells appears to confirm their active participation in the secretion of hydrochloric acid, which could explain the presence of neutral mucopolysaccharides in the apical region of the epithelial cells and positive immunoreactivity of the glandular cells towards H+/K+ -ATPase (Wilson and Castro 2011). The presence of mucous protection against the effect of the acid environment of the stomach as well as gastric proton pump genes is often used in research as an indication of the presence or absence of the stomach in fish (Smolka et al. 1994; Gawlicka et al. 2001; Jaroszewska et al. 2008). It does not seem that the glands present in the stomach of *P. semilunaris* were secretively inactive structures as Kobegenova and Dzhumaliev (1991) have stated with respect to the glands of all Gobiidae fish. It is possible that the gastric glands in *P. semilunaris* may present a lower level of gastric function, which was also suggested by Wilson and Castro (2011) with respect to alveolar gastric glands of other fish. This low level of gastric function and consequently the lesser importance of the stomach as a digestive organ would explain why the stomach is not visible at the gross anatomy level; however, further research into this issue is necessary.

### Proliferation of the stomach epithelium cells

The immunohistochemical research of proliferating cells nuclear antigen (PCNA) showed that the proliferating cells were scattered within the surface epithelium of the stomach and occasionally also in the evacuating section of the alveolar glands. A similar location of the progenitor cells was observed in *Zoarces viviparous*, with the difference that in *Z.* *viviparous*, the cell proliferation was visible in the wall of the entire follicular gland which in *P. semilunaris* was not observed (Wilson and Castro 2011). The presence of the proliferating cells in the evacuation duct of the alveolar glands in *P. semilunaris* recalls of the location of these cells in the tubular gastric glands. Wilson and Castro (2011) showed that in fish with tubular gastric glands, the multipotent stem cells are located in the neck region of the gastric glands, hence in the place of evacuation of the glands into the gastric pits. It is worth adding that restriction of the proliferation to the neck area is characteristic for adult fish, while lack of polarisation of the epithelium with respect to proliferation is typical for the larval forms (Rombout et al. 1984; Takashima and Hartenstein 2012). This suggests that the epithelial cells of the stomach of the adult specimens of *P. semilunaris* may have certain features of the epithelial cells of embryonic type, which allow quick adaptation of the alimentary canal to the changing food and environmental conditions. However, further studies are needed in this area, especially in regard to the structural plasticity of the alimentary tract in *P. semilunaris.*

### Oesophagus and oesogaster

The oesophagus of freshwater tubenose goby was not significantly different from the plane of the histological structure of this section in other fish species (Wilson and Castro 2011). The mucosa of the oesophagus of *P. semilunaris* as in other teleosts was without muscularis mucosa which did not allow the submucosa to separate (Islam 1951; Raji and Norouzi 2010). As in other freshwater Teleostei, the oesophagus of *P. semilunaris* was lined with stratified squamous epithelium provided with many large goblet cells. The dominant cells among the goblet cells were those producing a mixture of acid and neutral mucopolysaccharides which were also observed in the oesophaguses of other species of Gobiidae, such as *M. batrachocephalus*, *N. melanostomus*, *N. ratan* and *R. giurinus* (Kobegenova and Dzhumaliev 1991; Hur et al. 2005), except for the racer goby *B. gymnotrachelus* (Jaroszewska et al. 2008) in which the mucous cells were observed producing exclusively acid mucopolysaccharides. According to many scholars, the differentiation of chemical properties of the mucous is closely correlated with the function performed by them in the oesophagus (Sarasquete et al. 2001; Diaz et al. 2008). Acid mucopolysaccharides increase the viscosity of the mucus and mucosa protection of the oesophagus against bacterial and viral infections (Diaz et al. 2008; Ya et al. 2009). They may also influence the effect of neutral mucosubstances by creating a suitable chemical environment for digestive functions (Oliveira-Ribeiro and Fanta 2000; Abd El Hafez et al. 2013). The neutral mucopolysaccharides by surrounding the food facilitate its transport into the stomach and protect the wall of the oesophagus against mechanic damage. They probably participate in the enzymatic digestion of the food and in transforming it into chyme (Grau et al. 1992; Murray et al. 1994; Domeneghini et al. 1998; Diaz et al. 2008; Abd El Hafez et al. 2013). The role of the mixed type mucus has not been explored in detail so far. Due to the fact that the cells producing a mixture of acid and neutral mucosubstances were observed mainly in the oesophagus on young fish (Sarasquete et al. 2001; Gisbert et al. 2004), it is believed that their presence is a certain type of mechanism which allows the oesophagus to respond to various environmental changes (Domeneghini et al. 1998; Sarasquete et al. 2001). Although the researchers focused on the young fish, it cannot be excluded that a similar mechanism occurs in adult forms of some other fish species. Perhaps this mechanism in combination with a considerable potential to divide of the epithelial cells in the oesophagus of *P. semilunaris* deriving from the fragmentation of the proliferating cells along the entire length of the epithelium enable adaptation of these fish to new environmental food conditions.

The transitional zone situated between the oesophagus and the stomach of freshwater tubenose goby presented a clear abrupt transition from stratified squamous epithelium to simple columnar epithelium, as was described for *Pleuronectes americanus, Pleuronectes ferruginea* (Murray et al. 1994), *Trichomycterus brasiliensis* (Oliveira-Ribeiro and Fanta 2000), *Monopterus albus* (Dai et al. 2007) and *B. gymnotrachelus* (Jaroszewska et al. 2008). In many fish species, such as *Mystus gulio* (Kamal Pasha 1964) and *Clarias gariepinus* (Ikpegbu et al. 2013), a change in the epithelium is not however so clear cut and it occurs gradually. As a result of the differences between the histological structure of the wall of this region as well as the lack of a clear border between the oesophagus and the stomach, there are many discrepancies in the literature concerning the classification or name of this region. Many authors describe this region as part of the oesophagus, especially with regard to sea fish (Abaurrea-Equisoain and Ostos-Garrido 1996; Domeneghini et al. 1998; Abd El Hafez et al. 2013). Some authors refer to it as oesogaster (Kamal Pasha 1964; Ezeasor 1984; Grau et al. 1992; Arellano et al. 2001; Ikpegbu et al. 2013), and the term oesogaster is also applied to identify the transition region between the oesophagus and small intestine of fish without a stomach (Bremer 1978; Becker et al. 2010). Regardless of the manner of identification of this region, it is a short section of the alimentary canal whose wall contains elements occurring in the oesophagus as well as in the stomach.

## Conclusions

The study clearly shows the functional differentiation of the oesophagogastric segment of *P. semilunaris* despite the fact that the fish does not have an anatomically separate stomach. The stomach is a straight bag-like organ, which has the same anatomy in all areas. Between the oesophagus and the stomach, a short transitional zone is developed only. The histological indicators of the existence of the functional stomach are the presence of the following: (1) neutral mucopolysaccharides in the surface epithelial cells, (2) a well-developed layer of gastric glands in the lamina propria. This finding suggests higher adaptation of the species to utilise protein-rich food compared to fish lacking a stomach. Moreover, numerous proliferating cells were scattered throughout the surface epithelium, showing that the fish possess a potential to adapt the alimentary canal quickly to changing diet. The open question is how important is the presence of the functional stomach is in digestion processes and therefore in establishment of the species in invaded environments. Its role is expected to be limited as capacity of the stomach is small, which is associated with the lack of anatomical distinction.
